# Understanding *Cannabis sativa* L.: Current Status of Propagation, Use, Legalization, and Haploid-Inducer-Mediated Genetic Engineering

**DOI:** 10.3390/plants11091236

**Published:** 2022-05-02

**Authors:** David Charles Simiyu, Jin Hoon Jang, Ok Ran Lee

**Affiliations:** 1 Department of Applied Plant Science, College of Agriculture and Life Science, Chonnam National University, Gwangju 61186, Korea; davidmsimiyu@gmail.com (D.C.S.); jinhun92@naver.com (J.H.J.); 2 AgriBio Institute of Climate Change Management, Chonnam National University, Gwangju 61186, Korea; 3 Interdisciplinary Program in IT-Bio Convergence System, Chonnam National University, Gwangju 61186, Korea; 4 Botany Department, College of Natural and Applied Sciences, University of Dar es Salaam, Dar es Salaam P.O. Box 35091, Tanzania

**Keywords:** *Cannabis sativa*, hemp, haploid induction, bioengineering

## Abstract

*Cannabis sativa* L. is an illegal plant in many countries. The worldwide criminalization of the plant has for many years limited its research. Consequently, understanding the full scope of its benefits and harm became limited too. However, in recent years the world has witnessed an increased pace in legalization and decriminalization of *C. sativa*. This has prompted an increase in scientific studies on various aspects of the plant’s growth, development, and use. This review brings together the historical and current information about the plant’s relationship with mankind. We highlight the important aspects of *C. sativa* classification and identification, carefully analyzing the supporting arguments for both monotypic (single species) and polytypic (multiple species) perspectives. The review also identifies recent studies on suitable conditions and methods for *C. sativa* propagation as well as highlighting the diverse uses of the plant. Specifically, we describe the beneficial and harmful effects of the prominent phytocannabinoids and provide status of the studies on heterologous synthesis of phytocannabinoids in different biological systems. With a historical view on *C. sativa* legality, the review also provides an up-to-date worldwide standpoint on its regulation. Finally, we present a summary of the studies on genome editing and suggest areas for future research.

## 1. Introduction

**Figure 1:**
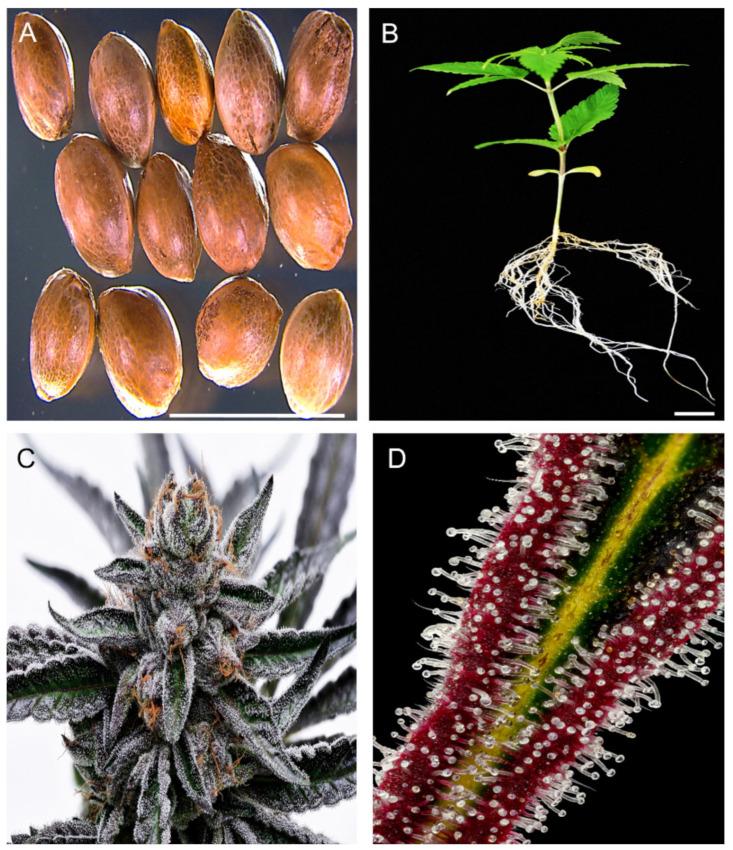
***Cannabis sativa* description.** (**A**) Hemp seeds of the Korean cultivar, Cheungsam. (**B**) Two-week-old hemp seedling. (**C**) Cannabis inflorescence and upper leaves covered with trichomes. (**D**) Glandular trichomes of Cannabis. (**C**,**D**) by Curtis Taylor and used here with permission. (Scale bar: 1 cm).

*Cannabis sativa* L. (Cannabis, hemp or marijuana) is an erect annual herb (Figure 1) of the *Cannabiceae* family [1]. Cannabis is considered to be monotypic (occurs as a single species), but arguments for its polytypic nature also exist [2]. The origin point of Cannabis is believed to be in Asia, with some evidence pointing to Central Asia [3], while other evidence points to the lower latitudes of the Asian continent [4] as the likely point of origin. However, it was in East Asia where humans first domesticated Cannabis more than 12,000 years ago [5]. Domestication of Cannabis resulted from understanding of the benefits obtained from different parts of the plant (Figure 1A-D). The multiple benefits of the plant, especially its use as a source of fibers, propelled the spread of the plant to other continents [6]. Today, Cannabis is popular for its medicinal and narcotic uses, which are attributed to its various secondary metabolites such as terpenoids, flavonoids, sterols and phytocannabinoids. The abundant phytocannabinoids it produces have made Cannabis one of the popular medicinal plants and has been used as medicine for centuries [7]. On the other hand, its narcotic effects caused the plant to be subjected to many decades of worldwide strict regulations. These restriction laws stalled its research for many years. However, the world is now witnessing an increase in relaxation of Cannabis laws [8]. Subsequently, interest in studies to decipher the full potential of the plant has also increased. For example, studies to synthesize phytocannabinoids in heterologous systems such as tobacco and fungi [9] have gained pace in recent decades. Due to the multiple usages of Cannabis, efforts to optimize its use will go hand to hand with deciphering the fast and efficient ways to fix desirable characteristics. This is why many studies are now directed at understanding the optimal conditions for Cannabis regeneration using different propagation methods. However, recalcitrant nature of Cannabis to indirect regeneration through tissue culture [10] has been a bottleneck for other studies in the plant, such as those involving genetic engineering. Nevertheless, researchers have recently started to unlock stable transformation methods for Cannabis [11,12], opening possibilities towards genetic engineering of the plant for more useful traits and products.

In this review, we bring together the opposing arguments for monotypic and polytypic models of Cannabis classification, as well as bring into focus the recent evidence that proposes shifting the Cannabis center of origin from Central Asia to lower latitudes of the continent. The review also highlights the methods used for Cannabis propagation and provides recent reports on Cannabis regeneration through tissue culture procedures, which have been challenging to researchers for many years. With a historical perspective, the review also brings together evidence and trends in Cannabis domestication and spread and provides a timeline review of Cannabis criminalization while giving an up-to-date report on the current legal status. Additionally, the multiple uses of the plant including the emerging ones, such as the use for bioenergy production, are highlighted while carefully comparing Cannabis prospects with other used plants. In the final parts of the review, we provide information on the current state on phytocannabinoid biosynthesis in heterologous systems and bring forth the new information on successful stable transformation studies, as well as provide the status of doubled haploid research in Cannabis.

## 2. Cannabis Taxonomy

### 2.1. Monotypic and Polytypic Models of Cannabis Classification

For breeders, accurate identification of the subspecies, or cultivar, is of paramount importance. In Cannabis, accurate classification has been at the center of taxonomists’ debate for centuries. However, the monotypic model of Cannabis classification is widely accepted among most botanists today. The monotypic classification model argues that there is only one species of Cannabis with two subspecies, *Cannabis sativa* subs. *sativa* (hemp) and *Cannabis sativa* subs. *indica* (marijuana) [13]. The monotypic classification of Cannabis supports the idea of Carolus Linnaeus who viewed *Cannabis sativa* as the only species from the genus *Cannabis*. Using chemical descriptors, Small and Cronquist [13] further classified the two subspecies into four distinct varieties, namely;

*Cannabis sativa* subsp. *sativa* var. *sativa* (hemp variety with domesticated characteristics);
*Cannabis sativa* subsp. *sativa* var. *spontanea* (hemp variety with wild characteristics);
*Cannabis sativa* subsp. *indica* var. *indica* (marijuana variety with domesticated characteristics);
*Cannabis sativa* subsp. *indica* var. *kafiristanica* (marijuana variety with wild characteristics).

Molecular studies conducted in recent decades have helped solidify the single-species model of Cannabis classification. McPartland and Guy [14], using barcode gaps in five different gene sequences, concluded that the molecular differences between Cannabis subspecies was too low to classify them as two distinct species (mean barcode gap of 0.41 ± 0.26) as the minimum barcode gap between plant species is between 1.3–5.7%, depending on the sequence used [15]. Additionally, the mean barcode gap of the two subspecies was even lower than that of other five known subspecies or varieties, indicating that hemp and marijuana cannot be regarded as separate species [14]. Another study in support of the monotypic model of Cannabis classification found the chloroplast genome of hemp to be very similar to that of marijuana with 99.99% sequence similarity and concluded the two to be a single species [16]. Furthermore, comparative sequencing of selected chloroplast and mitochondrial DNA loci in Cannabis individuals from different parts of the world suggested hemp and marijuana separate at a rank lower than that of species [17]. Additionally, a study by Zhang et al. [4] which analyzed five chloroplast DNA regions of 645 individual from 53 different Cannabis accessions, also concluded that the plant occurs in one species typified by *C*. *sativa*. The study further suggested that the species be subdivided into three subspecies, namely, subsp. *sativa*, subsp. *indica* and subsp. *ruderalis*. 

Despite the monotypic model of Cannabis classification being widely accepted, a polytypic model of its classification has also been suggested. The polytypic model of Cannabis classification suggests existence of multiple species in the *Cannabis* genus. Diversions from the one-species concept began with observations by the French botanist Jean Baptiste de Lamarck. Using morphological and chemical descriptions, Lamarck divided the genus into two species, namely *Cannabis sativa* and *Cannabis indica* Lam. [18]. Lamarck observed that *C. indica* differed from *C. sativa* in stalks, branching habit, leaflets, odor and psychoactive nature [1]. Other scientists after Lamarck have noted several other differences that suggests Cannabis occurs in multiple species. Hillig and Mahlberg [19] for example, studied the difference in cannabinoid level between 157 Cannabis accessions from different geographic origins and concluded that Cannabis should be divided into two species, *C. sativa* and *C. indica.* Moreover, other scientists argue for classification of Cannabis into three distinct species after a third Cannabis species (*Cannabis ruderalis* Janisch.) was proposed by D.E Janischewsky in 1924 [18]. This three-species model of Cannabis classification was also supported by Anderson [20] based on leaf morphology. 

Contrary to the monotypic model which has been supported by morphological, chemical and molecular studies, the polytypic model has mainly been supported by morphological and chemical descriptors. The only study found, that suggests a multiple species classification of Cannabis based on molecular description, was carried out by Hillig [1]. In studying allozymes variation in Cannabis accessions from all the continents, Hillig [1] analyzed 52 alleles from 11 different enzymes and concluded that the accessions came from three distinct gene pools. These were the *C. sativa* gene pool (plants from Europe, Asia Minor and Central Asia), *C. indica* gene pool (from Afghanistan, Pakistan, India and Nepal) and *C. ruderalis* from Central Asia. However, the *C. ruderalis* gene pool was considered less certain due to the limited number of its accessions analyzed. Despite the appearance of three distinct gene pools, Hillig’s study did not provide evidence that the amount of observed genetic variation in the analyzed alleles are enough to separate Cannabis into more than one species.

### 2.2. Identification of Cannabis 

Practical and accurate differentiation of the two subspecies of Cannabis is imperative in fields such as agriculture, law enforcement, as well as in industries such as food and pharmaceutical. Authorities in Europe, America and other members of the United Nations use cannabinoid quantity as an identification method. This method of Cannabis classification was first used by Small and Cronquist [13], wherein a concentration of 0.3% (dry weight) of Δ^9^-tetrahydrocannabinol (THC) is proposed to be a chemical demarcation between marijuana and hemp. Plants with THC concentration of <0.3% are considered as hemp (fiber-type) while those containing THC of ≥0.3% are considered as marijuana (drug-type) [13,21]. Besides using phytocannabinoids, monoterpenoids and sesquiterpenoids content can also be used to distinguish hemp from marijuana [22]. Variations in terpene synthase genes is also associated with the differences between drug-type Cannabis cultivars [23]. The monoterpene (myrcene) content strongly correlates with cultivars having an ‘earthy’ aroma, and sesquiterpenes (bergamotene and farnesene) concentration correlate with cultivars having a ‘sweet’ or ‘herbal’ aroma. Moreover, Grassi and McPartland [24] discovered that the ratio of THC to cannabidiol (CBD) is genetically controlled (monogenic) and proposed another method of Cannabis classification, that is, cannabinoid quality. Cannabinoid quality is a more stable aspect than cannabinoid quantity, the latter being prone to changes as a result of factors such as environment and age of the plant [25]. Hillig and Mahlberg [19] used the cannabinoid quality to divide Cannabis into three chemotypes (type I, type II and type III). These chemotypes are determined using a quotient obtained mathematically by a formula Log_10_ (THC%/CBD%). Plants with quotient >1.0 are classified as type I, those with a quotient value <−0.7 as type II and those with quotient values between -0.7 and 1.0 as type III plants [19]. Moreover, two other chemotypes have been suggested. These are chemotype IV, with cannabigerol as a dominant cannabinoid [26], and chemotype V which has no detectable cannabinoids [27]. Furthermore, an easy-to-read cannabinoid quality quotient has been developed by modifying the Hillig and Mahlberg classification. In this cannabinoid quality, Cannabis is divided into two groups by plotting results of the measured CBD and THC concentration as Log_10_ (CBD/THC) [28]. Plants with values <0.0 were assigned to type I and those with values >0.0 were grouped together as type II/III. 

Several studies have been conducted to determine the genetic variation between hemp and marijuana [29]. Genetic markers such as the randomly amplified polymorphic DNA (RAPD) [30,31], amplified fragment length polymorphism (AFLP) [32], single-nucleotide polymorphisms (SNPs) [21,33,34,35], single-tandem repeats (STRs) [36], simple sequence repeats (SSRs) [37], have been successfully used to distinguish the two subspecies of Cannabis as well as varieties within subspecies. 

The criteria used to classify Cannabis have sometimes seen an overlap of subspecies [34]. For example, based on cannabinoid quantity, a Korean hemp cultivar Cheungsam, which is cultivated for fiber production and has THC content above 0.3% [35], can be classified as marijuana. The best approach therefore is not to rely on one method for identification, but to apply several methods that will help understand the chemical, genetic and physical nature of the plant breeders want to work with.

Breeding of crops with suitable traits requires also understanding of the environment where a variety is adapted to. The origin, and the spread history of the variety of interest, often offers a clear way for understanding the suitable environment for its optimum growth and development.

## 3. Origin and Distribution

**Figure 2:**
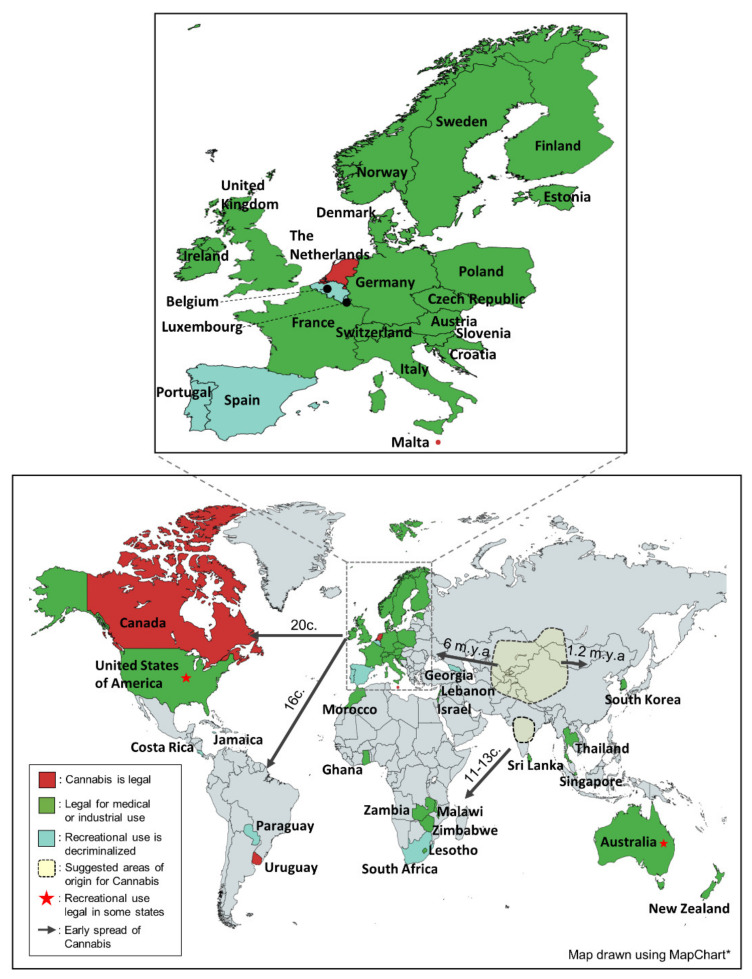
**Legal status and early spread of Cannabis.** From its proposed centers of origin (Central and Southern Asia), Cannabis spread to the other continents of the world. Today, Cannabis use for medical purposes is permitted in many countries, particularly in Europe and North America. Recreational Cannabis is permitted in Canada, The Netherlands, Malta and Uruguay while it is decriminalized in Portugal, Spain, Luxembourg, Belgium, Georgia, Costa Rica, Paraguay, Jamaica and South Africa. The recreational use of Cannabis is also permitted in some states of the US and Australia. A close-up part of Europe is shown in an insert map, 5.5× magnified. (*c.: century, m.y.a.: million years ago*, * https://mapchart.net/world.html (accessed on 10 March 2022)).

It has been widely suggested that Cannabis originated in the Central Asia region (Figure 2). However, there has not been a consensus on the exact location of its ancestry. Several locations within the Central Asia region have been proposed to be the likely origin points of Cannabis [38]. Specifically, a study on the Cannabis subfossil pollen deduced that the plant originated in the Qinghai Lake area of Northeastern Tibet [3]. Being the biodiversity center of both Cannabis and *Humulus* [39], the Central Asia region is considered as the most likely origin point of the two plants. However, the prevalent Central-Asia-origin hypothesis of Cannabis origin is not the only one proposed. Molecular studies carried out by Zhang et al. [4] indicated Cannabis originated in the low latitudes of the Asian continent. Assessing chloroplast DNA of 645 Cannabis individuals from high, middle, and low latitudinal regions of the Asian continent, it was concluded that lineages inhabiting the low latitude regions of Asia (Figure 2) were the earliest to diverge in Cannabis’s evolutionary history. Cannabis origin was therefore suggested to be the regions of India and/or Southeastern Asia [4]. Despite scientific evidence suggesting several Cannabis origin areas, there exists a consensus that ascertaining the exact point of origin is difficult. This is due to two major reasons. One is that Cannabis has been used by humans for millennia, hence subjected to human selection in a way that has extensively altered the plant. This implies that true wild-type Cannabis, through which true ancestry could be traced, does not exist [5,38]. Second, the fossilized pollen grains of Cannabis are indistinguishable from those of *Humulus lupulus* [3,40], making it difficult to correctly identify and use fossilized pollen grains to ascertain ancestry of the two species. 

Despite the lack of agreement about the exact area of origin, history of the early and current distribution of Cannabis is well understood. In a study of Cannabis chloroplast DNA, it was postulated that Cannabis and *Humulus* diverged from a common ancestor before 18.23 million years ago [4]. More recently, molecular studies have suggested that the ancestral Cannabis effective population size reached its peak ca. 1 million years ago [5]. This suggestion was congruent with the conclusions made by Zhang et al. [4] who inferred the crown age of Cannabis to be at 2.24 million years ago. Nevertheless, by this time the plant had already evolved and widely distributed in the Eurasian region. Archeological studies postulated that the plant spread from its center of origin first towards Europe about 6 million years ago and then towards Eastern Asia about 1.2 million years ago (Figure 2) [3]. Humans have played a major role in the global distribution of the plant in recent history. The millennia-long use and domestication of Cannabis suggests the spread of Cannabis from Asia to other continents was human-influenced [41]. Achene and pollen fossils as well as ancient artifacts have indicated humans have been collecting Cannabis since ca. 12,000 years ago in Asia and more than ca. 8000 years ago in Eastern Europe [5,42]. The multiple uses of the plant, particularly in fiber making, propelled its early distribution to different locations. Fiber remains in the Southeastern area of Asia that date back to ca. 4000–3000 years ago have been found [42]. This indicates that hemp and the fiber-making practice found its way to this region of the Asian continent in later years. Entry of Cannabis in other continents (Figure 2) was influenced by inter-continental trade and colonialism. In Africa, Cannabis is believed to have entered from India through the East African coast between the 11th and 13th centuries [5,43]. Early in the 19th century, European explorers in the African continent found Cannabis was used extensively by locals [43]. Moreover, the spread of Cannabis to American continents coincided with the coming of European colonists who introduced the plant as a cash crop. In South America, for example, Cannabis farming was introduced in 1545 by the Spanish while the English monarchy introduced hemp to their colonies in Canada (1606), and Virginia (1611) [6,7]. Drug-type Cannabis reached North America early in the 20th century [5].

## 4. Cannabis Cultivation 

### 4.1. Methods of Cannabis Propagation

Cannabis domestication began more than 12,000 years ago in East Asia [5]. Initially, Cannabis was used as a multipurpose plant until ca. 4000 years ago when selection for either drug or fiber production began [5]. Currently, Cannabis cultivation takes place in both outdoor and indoor farming systems. Choice of the cultivation site depends much on the desired end-product. Less valuable products such as fibers and seed oils are usually cultivated in open farms [44]. Farming for high-value Cannabis products, such as those used for medicinal or recreational purposes, may also be conducted outdoors. However, to maximize quality and quantity of the plant’s final products, greenhouses or indoor production facilities are usually used [44]. 

**Figure 3:**
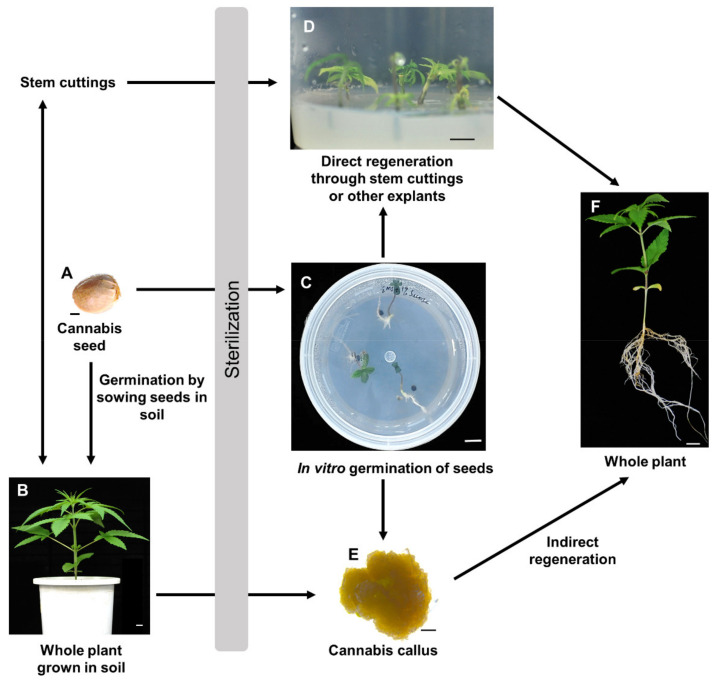
**General procedure of Cannabis propagation.** Germination of (**A**) Cannabis seeds can take place (Touret) in soil, as well as in sterile and optimal conditions to make (**C**) in vitro grown seedlings. Direct regeneration of Cannabis can be achieved through (**D**) stem cuttings in sterile conditions. It can also be achieved through culturing of the excised hypocotyl, cotyledon, and true leaves of (**C**) in vitro grown cannabis seedlings. The stem cuttings from a cannabis plant can also be used by breeders to cultivate multiple clones of the mother plant (B) in soil. Indirect regeneration of cannabis through (**E**) callus has been achieved by few researchers. The cannabis callus cells can be obtained by culturing explants from (**B**,**C**). Upon acclimatization procedures, in vitro grown seedlings will grow to (**F**) whole plant. Arrowhead shows direction of the propagation method. Scale bars: 2 mm (**A**,**E**), 1 cm (**B**–**D**).

Cannabis can be propagated in open or closed farming systems using seeds, through clonal propagation or tissue culture methods (Figure 3). In the indoor controlled farming systems, clonal propagation is the more preferred propagation method, usually performed through stem cuttings (Figure 3D) [10,44,45]. By propagating the successful mother plant, desired qualities are maintained for future generations [46]. However, several limitations of clonal propagation have been reported. These include the need for large space to maintain the desired mother plants, high possibility of infections and pests, as well as difficulty in maintaining a vegetative state for auto-flowering varieties [44]. 

### 4.2. In vitro Cannabis Regeneration

To minimize the limitations of clonal propagation, tissue culture methods are alternatively used. Several studies in the past decades have been conducted to determine the optimal conditions for tissue culture propagation of Cannabis [47,48]. Most in vitro studies have achieved successful callus induction (Figure 3E) of various Cannabis explants using different combinations of cytokinins and auxins [10,44]. In vitro direct regeneration from hypocotyls, cotyledons and leaves from 7-day-old Cannabis seedlings has also been achieved recently by Galán-Ávila et al. [11]. The study reported hypocotyls as the best responding explant with higher rate of regeneration (53.3%). However, studies on indirect Cannabis regeneration through callus tissues are conflicting. Some studies have reported successful regeneration from callus tissues [12,49,50,51,52,53,54]. However, some reports indicate lack of reproducible and robust protocols for indirect regeneration of Cannabis [10,44,55]. Nevertheless, a recent study reported a successful indirect regeneration from calli of different explants of hemp [12], with the highest shoot regeneration rate of 7.1% achieved from calli induced from embryo hypocotyl of immature grains. Additionally, the regeneration efficiency increased 1.7 times by the overexpression of genes responsible for shoot organogenesis [12]. Despite these successes, more studies need to be carried out to establish higher indirect regeneration rates for both hemp and marijuana. Studies to understand optimal conditions for in vitro growth of Cannabis are equally important. Conditions such as sucrose concentration, light intensity and light spectrum have been found to influence different aspects of Cannabis growth including shoot length, root length, shoot number, number of nodes and canopy area [56].

## 5. Cannabis Use

### 5.1. A Multipurpose Plant 

**Figure 4:**
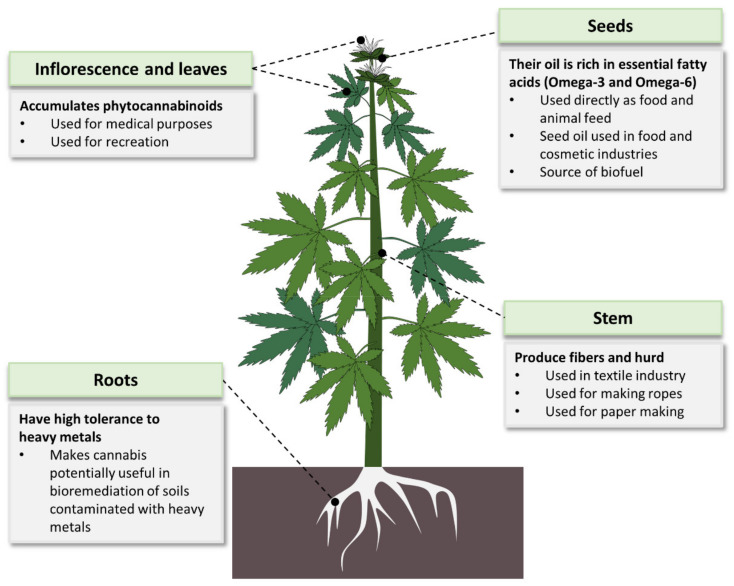
**Cannabis usage:** Almost all parts of the Cannabis plant can be used in one or more ways. The plant’s seeds are highly nutritious while its flowers accumulate medicinal secondary metabolites (phytocannabinoids). Cannabis stem is used for producing quality fibers, and its roots are efficient in bioremediation.

Cannabis is mostly known for its therapeutic and narcotic effects. However, it has many other applications (Figure 4). It is used in various industries including food, cosmetics, energy and textiles [7,18,57,58]. Studies have also indicated a potential use of Cannabis in bioremediation of contaminated soils. Cannabis showed high tolerance to heavy metals such as cadmium [59,60,61], copper and nickel [60,61], as well as effective absorption of atrazine from the soil [62], making the plant an efficient bioremediation agent for heavy metals. 

One of the widely used product of Cannabis is its fibers. In fact, for thousands of years the plant was more valued for its production of fibers than for any other use [41]. Cannabis fibers are made from the plant’s stem. The bast (bark) of Cannabis gives longer fibers than those from its hurd [63]. Cannabis fibers are used in various products such as textiles, ropes, canvas, home furnishing and industrial products [63]. Cannabis fibers can also be used to make paper products. The paper industry heavily relies on trees as the major raw material, causing alarming environmental degradation [64,65], indicating the urgent need for alternative sources. Some of the non-wood paper sources used include hemp, sugarcane bagasse, flax, jute, bamboo and cereal straws. Among these, hemp is considered the best option. This is because hemp grows well in both temperate and tropical areas and has a higher yield per acre than the other alternatives (flax yield is half that of hemp) [65]. Hemp is also friendlier to the environment and produces better quality paper than tree sources [64]. In the early 2000′s, hemp fibers lost their place as the main Cannabis product after the global hemp seed production overtook that of hemp fibers [66]. Cannabis seeds (Figure 1A) are used directly as food, animal feed and in production of seed oil for food and cosmetic use. Mankind has known the nutritive value of Cannabis seeds and has used them as food for many years. For example, in Asian societies, Cannabis seeds have been an important diet for many generations [7]. One of the important nutritive values of Cannabis seeds is the abundance of two essential fatty acids (EFAs) linolenic acid (omega-3) and linoleic acid (omega-6) [58]. The ratio of omega 6 to omega-3 in Cannabis seeds is 3:1, which is optimal for human health [58,67]. Additionally, Cannabis is ranked among the top known crops rich in the EFAs [63,67]. Cannabis seeds are also abundantly endowed with all (including essential) amino acids; fibers; vitamins A, D, E and C; thiamine; and riboflavin as well as minerals such as phosphorus, iron, potassium and calcium [67]. As a result of their rich nutrients, Cannabis seeds and their oil have continued to be in the food and cosmetics markets of Asia, Europe and America [18]. According to the World’s Top Exports report, countries spent a total of USD 3.1 billion to import Cannabis oils in 2019 with the top three importers of Cannabis oils being the United States of America, Germany and South Korea, with the latter spending USD 207.7 million [68]. Moreover, the total legal Cannabis market has been on an increasing trend for the past few years [63]. Projections predict this market to continue increasingly, reaching an estimated value of USD 27.89 billion by 2024 [69].

Cannabis is also a good source of biofuels [57,70,71]. When compared to oil seed rape (OSR) and sugar beet (crops used for bioenergy production in Europe), hemp is more efficient in reducing greenhouse gases emission [70]. Additionally, hemp is not extensively used as a food crop, meaning its use as a biofuel does not directly endanger food supply [70]. Hemp has therefore been concluded to be a better substitute of fossil fuels than biodiesel and bioethanol from OSR and sugar beet, respectively [70]. Moreover, a study to compare the bioenergy potential of different crops ranked Cannabis higher in terms of per-hectare revenue generation than kenaf, switchgrass and sorghum [71]. 

The use of Cannabis as an intoxicating agent has been the main reason for its criminalization, but the practice of smoking Cannabis is believed to have been in existence for thousands of years. Excavation of wooden burners containing Cannabis remains in China’s ancient tombs [72] offered an insight on the ancient narcotic use of Cannabis. These excavations indicate Cannabis smoking was practiced in human societies about 2500 years ago [72]. Although it is not yet clear whether the ancient societies smoked Cannabis for recreation or as part of a sacred ritual, modern societies widely smoke Cannabis for recreation. Cannabis is reported to be the most used illicit drug in the world [73]. The ongoing changes of Cannabis’s legal status and increase in the world population make it unlikely for the non-medical use of Cannabis to decrease. This speculation is also supported by the finding that recreational Cannabis users increased by 30% in the past two decades [73].

Value addition of Cannabis and its products will have to include the enhancement of suitable traits of the plant. With the plant having multiple uses, breeders have multiple options to improve Cannabis quality. For example, enhancing quality of Cannabis fibers can involve making plants with less lignified stems. This can be achieved through genetic engineering methods as genes responsible for lignin formation, such as the patatin-related phospholipase As [74,75,76,77], have been elucidated in recent years. Similar genes can be manipulated to improve the bioenergy potential of Cannabis because with less lignin, cellulose becomes more accessible for biofuel use [78].

### 5.2. Cannabis as Therapeutic Agent

Cannabis has been used in folk medicine for centuries [7]. Likewise, pharmaceutical industries in recent decades have rolled out several Cannabis-based medicines. These medicines are made from CBD extracts, THC extracts or a combination of several Cannabis metabolites (GW Pharmaceuticals). One example is the use of CBD for the treatment of epilepsy which was reported by the World Health Organization (WHO) to pass the clinical trials. Trials on animals and human subjects have shown effectiveness of CBD in stopping or reducing seizures including those caused by Dravet syndrome, a complex childhood epilepsy with high mortality rate [79]. The United States Food and Drug Administration (FDA) has approved one CBD-derived drug (Epidiolex) as a safe and effective treatment for seizures [80]. The FDA has also approved the synthetic-THC drugs Marinol, Syndros and Cesamet for treatment of nausea and anorexia associated with cancer chemotherapy and AIDS, respectively [80]. Moreover, the National Institute for Health and Care Excellence of the United Kingdom (NICE) published a guideline for use of CBD- and THC-based products for treatment of various conditions. These conditions include the treatment-resistant epilepsy (Lennox–Gastaut syndrome, Dravet syndrome), spasticity, as well as intractable nausea and vomiting [81]. Other conditions in which cannabinoids are used as a treatment or remedy are listed in Table 1. Nevertheless, Cannabis products approved for therapeutic use are few. Therefore, more research on the safety, effectiveness and efficacy of Cannabis use in other health conditions is required. 

### 5.3. Side Effects of Cannabis Use

Although Cannabis is known to be medicinal, it is also considered an illicit drug because its phytocannabinoid, THC, is highly psychoactive. Studies have indicated THC interferes significantly with brain development when used by young individuals [82]. THC is also associated with the development of psychiatric diseases such as schizophrenia later in life [82,83,84,85,86,87,88]. A summary of the conditions caused by the use of Cannabis are summarized in Table 1. Apart from the health conditions caused by Cannabis use, studies have also linked one of its cannabinoids to interference in sensory coordination. A study involving 22 healthy individuals implicated THC use in several sensory malfunctions [90]. These include altered perception, deficit in working memory, loss of spontaneity, distracted verbal fluency and psychomotor retardation, soon after isolated THC was intravenously administered. As a result of its potential to interfere with psychomotor performance, Cannabis use can be dangerous to drivers and can affect such functions that require excellent sensory coordination. A study involving 456 drivers in Norway, for example, found that higher blood THC concentrations (0.32–24.8 ng/mL) significantly impaired their driving skills [91].

## 6. Legal Status of Cannabis 

### 6.1. History of Cannabis Prohibition

**Figure 5:**
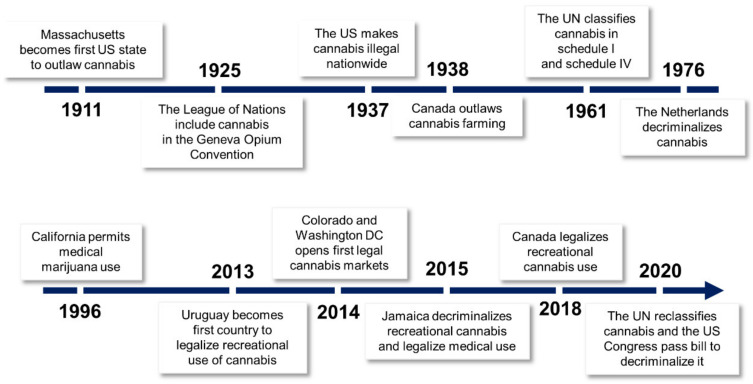
**Major events in the legal history of Cannabis.** The first six decades of the 20th century encompassed the enactment of strict laws to regulate Cannabis use. Soon after, the relaxation of regulations started with The Netherlands’ decriminalization of *Cannabis* in 1976. The first country to legalize recreational use of Cannabis was Uruguay in 2013, followed by Canada five years later. In 2020, the United Nations (UN) reclassified Cannabis by removing it from its most strict schedule of narcotic drugs.

Cannabis is a controversial plant. The narcotic aspect of the plant calls for continuation of its criminalization, but at the same time its beneficial aspects are difficult to neglect. The history of Cannabis criminalization began in the early decades of the 20th century (Figure 5). Fear of the plant’s psychoactive effects and intentions to prohibit its international trade prompted the international community to start discussing prospective Cannabis regulation [104]. As these efforts mounted, Cannabis was included in the League of Nations’ 1925 Geneva Opium Convention [8]. Although this event did not impose absolute restrictions on trade and use [105], it set a reference point for Cannabis regulation in different countries. For example, in the USA, the Marihuana Tax Act which made Cannabis illegal in all the states was enacted in 1937 [106]. 

**Figure 6:**
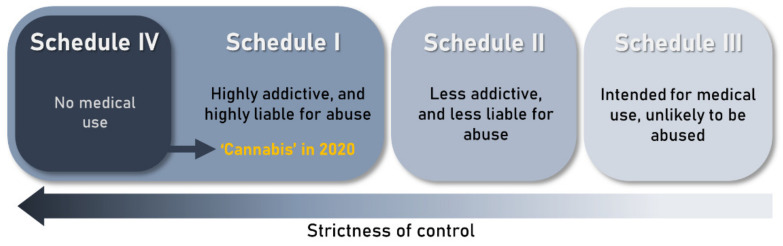
**Narcotic drugs scheduling system as classified by the Single Convention on Narcotic Drugs of 1961.** The Single Convention on Narcotic Drugs of 1961 classifies drugs and their preparations in four schedules based on their dependence potential, abuse liability and therapeutic usefulness [107]. Substances in schedule IV include the drugs in schedule I which have no therapeutic use. Following the recommendations of the World Health Organization, Cannabis was removed from schedule IV in 2020.

The worldwide prohibition of Cannabis was consolidated in the United Nations Single Convention on Narcotic Drugs in 1961 which grouped narcotic drugs into four schedules: I, II, III and IV (Figure 6). The convention listed Cannabis in two of the schedules with high restrictions, schedule I (substances that are highly addictive and liable to abuse) and schedule IV (substances that are highly addictive, liable to abuse and lack therapeutic value) [105]. Being listed in schedule IV meant that Cannabis use and trade for all purposes, including medical use, was to be prohibited. Although Cannabis is still widely criminalized, arguments have been raised for the review of its regulatory laws. With an increased understanding of the nature of Cannabis phytochemistry, countries began reviewing their regulation laws (Figure 5). The first country to legalize Cannabis use for medical and recreational use was Uruguay in 2013 [8]. Since then, more countries have followed suit (Figure 2). Optimism for global legalization of Cannabis, at least for medical use, gained momentum in 2019 following a report by the WHO, which was critical of the placing of Cannabis in the United Nations narcotic drugs scheduling system. The WHO concluded that listing Cannabis in schedule IV ignores the therapeutic value of the plant and hence should be rescheduled. The WHO recommendations were adopted by the United Nations’ Commission for Narcotic Drugs in December 2020 [107], meaning that Cannabis is currently listed only in schedule I of the scheduling system (Figure 6). Reclassification of Cannabis by the United Nations provides an important framework for nations reviewing laws on the regulation of Cannabis and its products.

### 6.2. Current Legal Status in the World

With the exception of a few countries (Figure 2), many countries regard Cannabis as illegal. However, the support for its legalization is growing. The USA House of Representatives, on December 4th, 2020, passed a bill to decriminalize Cannabis in the country. The bill has not yet been approved by the country’s Senate. Nevertheless, many States have changed their laws on Cannabis regulation. Currently, 36 out of the 50 USA States, including Washington DC, have legalized the use of Cannabis for different purposes. Of the 36 states, 18 states permit recreational use and the rest have allowed Cannabis use for medical purposes [108]. Many other countries have also revised their laws to allow Cannabis use for either recreational or medical purposes [8,109,110,111,112,113]. However, the recreational use of Cannabis has only been legalized in Canada, Uruguay, Malta and the Netherlands while a few other countries have only decriminalized its use (Figure 2). In some countries (not included in the map), possession of a small amount of Cannabis for personal use is tolerated. Nevertheless, in such countries (including Poland, Colombia and Croatia) law enforcement authorities can still prosecute with discretion [109]. 

## 7. Phytocannabinoids

### 7.1. Types and Biosynthesis

**Figure 7:**
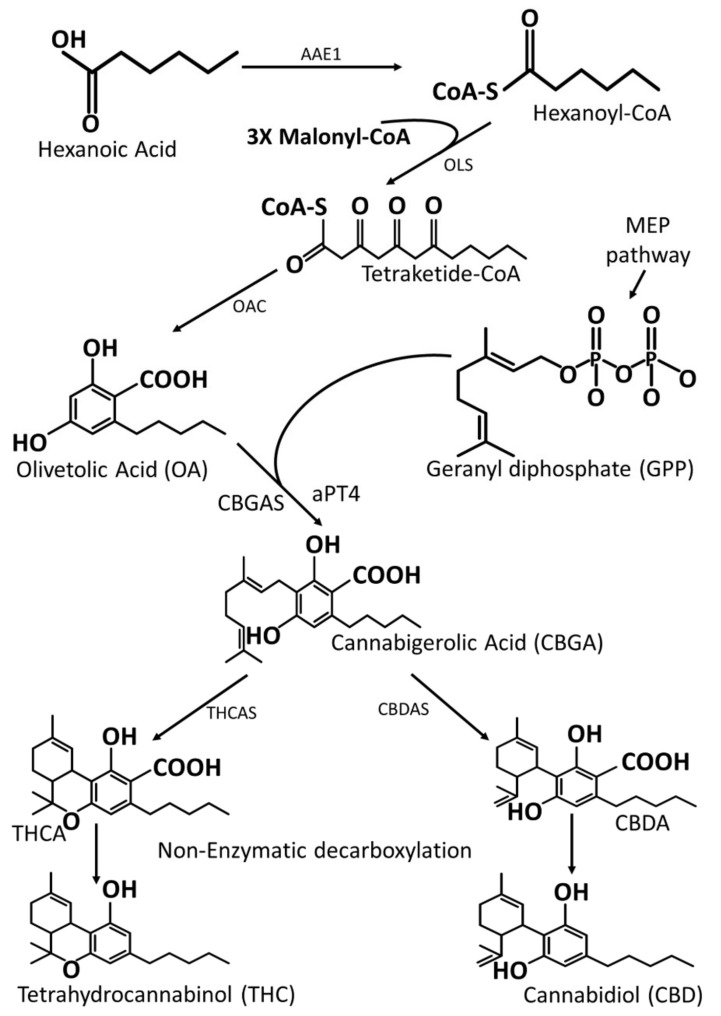
**A simplified overview of phytocannabinoids’ biosynthesis in Cannabis using THC and CBD as representative molecules.** The phytocannabinoid acids in Cannabis are enzymatically synthesized from one precursor molecule, CBGA. CBGA is derived from MEP and polyketide pathways under the mediation of CBGA and aPT4 enzymes. AAE1, Acyl-activating enzyme 1; OLS, olivetol synthase; OAC, olivetolic acid cyclase; CBGAS, cannabigerolic acid synthase; aPT4, aromatic prenyltransferase 4; THCAS, tetrahydrocannabinolic acid synthase; CBDAS, cannabidiolic acid synthase; THCA, tetrahydrocannabinolic acid; CBDA, cannabidiolic acid.

Cannabis synthesizes its phytocannabinoids in the glandular trichomes (Figure 1D) which are abundant in the bracts of female flowers (Figure 1C) [114]. At least 113 phytocannabinoids have been identified so far [115]. These include the prominent THC and CBD, which usually define the plant’s classification and use. The main precursor compound of all known phytocannabinoids is cannabigerolic acid (CBGA) which is synthesized from two compounds, olivetolic acid from the polyketide pathway and geranyl diphosphate (GPP) from the plastidial methyl erythritol phosphate (MEP) pathway [39,116]. The CBGA is synthesized within the disk cells found in glandular trichomes, and then transported to the storage cavity where phytocannabinoid synthases found in secretory vesicles catalyze synthesis of various phytocannabinoids [116]. The phytocannabinoids are synthesized in the form of acids [117]. THC, for example, is synthesized in the form of Δ^9^-tetrahytrocannabinolic acid (THCA) and CBD synthesized as cannabidiolic acid (CBDA) (Figure 7). They can be converted into their bioactive neutral forms through non-enzymatic decarboxylation processes normally through heating or long-time storage [118]. It is their neutral forms which allow phytocannabinoids to have biological interactions with cannabinoid receptors in the human cells [116,117]. 

### 7.2. Heterologous Biosynthesis of Phytocannabinoids

The revision of Cannabis restriction laws and the increased knowledge about its potential use in various industries has led to increased interest in cannabinoid synthesis. Research into both heterologous and in planta biosynthesis of this valuable group of metabolites has been going on for decades. However, most research has been directed at heterologous biosynthesis (Table 2), possibly because of the legal status of Cannabis, which make cannabinoid synthesis in other systems less problematic. Another factor that might explain interest in heterologous systems for cannabinoid synthesis is the genetic nature of the plant itself. Cascini et al. [119] quantified the THCA synthase gene in Cannabis and found no correlation between THCA synthase gene copy number and the amount of THC in Cannabis samples. Therefore, attempts to enhance THCA synthesis in Cannabis by overexpressing its gene seem futile. The first heterologous biosynthesis of a cannabinoid compound reported a successful THCA biosynthesis in a tobacco root’s system using cannabigerolic acid (CBGA) (Figure 7) as a precursor compound [120]. Although the amount of produced THCA was small (8.2% conversion rate of supplied CBGA), the study gave an impetus to further research. Three years later (in 2007), the same research group reported a novel expression system for THCA synthase using *Pichia pastoris* [121], wherein the enzyme showed a much more improved activity (98% conversion rate of supplied CBGA). Additionally, the *Pichia pastoris* system for the heterologous synthesis of THCA was found to be more efficient when compared to *Saccharomyces cerevisiae* system [122,123]. The use of *Escherichia coli* as a system for cannabinoid synthesis has been reported to be inefficient [122]. A recent and perhaps the most comprehensive study on heterologous cannabinoid synthesis was conducted by Luo et al. [124]. In the study, four major phytocannabinoids (Δ9-tetrahydrocannabinolic acid, cannabidiolic acid, Δ9-tetrahydrocannabivarinic acid and cannabidivarinic acid) were successfully biosynthesized in *Saccharomyces cerevisiae* from the simple sugar galactose. This study promoted the possibility of using other organisms for cannabinoid synthesis. Moreover, it has recently been reported that other intermediate compounds in the phytocannabinoids pathway can be synthesized in heterologous systems [9]. In this study, two intermediates, olivetolic acid and cannabigerolic acid (Figure 7), were synthesized using aromatic prenyltransferase (*CsaPT4*) in *S. cerevisiae*. Using the same gene, olivetolic acid glucoside was also synthesized in *Nicotiana benthamiana* [9]. Although successful synthesis of cannabinoids has been carried out in microorganisms and in planta heterologous systems, there are still challenges which need addressing to allow large-scale production. One such challenge was highlighted by [125], who reported that THCA and CBGA are cytotoxic in *C. sativa* and tobacco cells. To avoid the cytotoxic effects, Cannabis secretes and stores the phytocannabinoids into the storage cavity of the glandular trichomes (Figure 1D). Therefore, mass production of cannabinoids in heterologous systems has to be carried out in organisms that are structurally capable of avoiding cytotoxicity of these metabolites. Plants such as tomatoes and Artemisia have glandular trichomes, making them possible candidates for this endeavor. Additionally, there are valid concerns about whether the cannabinoids produced in heterologous systems would have similar effects possessed by the same cannabinoids produced in nature. This is because of what cannabinoid researchers have termed as the “entourage effect”, which occurs when specific phytocannabinoids work in synergy with other secondary metabolites to bring about their known effects [126,127]. It is therefore important that efforts to increase phytocannabinoid supply are more focused on enhancing their endogenous biosynthesis. One approach to enhance endogenous phytocannabinoid synthesis is through increasing trichome density. In *Artemisia annua,* for example, increased trichome density correlated with an increase in artemisinin [128]. One approach to increase trichome density in Cannabis has also been postulated [129]. This work includes the systems and methods for increasing trichome formation and density in Cannabis using two transcription factors, *MYB1* and *MYB12* from *A. annua* and *C. sativa*, respectively. Another promising method to increase trichome density is polyploidization. Leaves of tetraploid Cannabis plants were reported to have higher glandular trichome density than diploid Cannabis plants [130]. The same study also found a significantly higher amount of CBD in the buds of tetraploid plants, but the CBD content in leaves of tetraploid and diploid plants was not significantly different. The reported studies on enhancement in the biosynthesis of cannabinoids in heterologous systems and in planta show promising results. Nevertheless, more efforts are still needed to ensure supply of these valuable compounds meets the projected increase in their demand.

## 8. Genetic Engineering

### 8.1. Genome Modification/Editing Studies in Cannabis

Genetic engineering is a method that has been widely used to improve traits and to obtain new cultivars with desirable characteristics. To achieve this, breeders can use conventional breeding, genetic modification or genome-editing methods [131]. Genetic modification entails the transfer of genetic materials from one species to another (transgenic) or within the same species (cisgenic), whereas in genome editing a specific part of a gene can be edited using sequence-specific nucleases such as zinc-finger nucleases (ZFNs), transcription-activator-like effector nucleases (TALENs) and regularly interspaced short palindromic repeat-associated endonucleases (CRISPR/Cas) [131]. The use of CRISPR/Cas allows breeders to edit a gene sequence using an enzyme and a small guide RNA which can be removed afterwards, making the edited lines free of foreign genetic materials [10]. The molecular tools needed to achieve genome modification or editing can be delivered into the cell system by various methods, including *Agrobacterium*-, nanoparticle- and polyethylene glycol (PEG)-mediated transformation. In Cannabis, the aforementioned methods have been used in gene modification studies [48]; however, regeneration of the transgenic plants has remained a major challenge. For example, Cannabis callus was successfully transformed using *Agrobacterium tumefaciens* (strain EHA101) carrying a gene encoding phosphomannose isomerase, but regeneration of the transformed calli was not achieved [132]. In another study, *Agrobacterium rhizogenes* transformation of hemp hypocotyls resulted in transgenic hairy roots, with no report of regeneration of the transformed tissues [133]. A lack of replicable and reliable Cannabis regeneration protocols prompted more studies on transient gene transformation for studying gene functions. Virus-induced gene silencing (VIGS) was used for reverse genetic studies in hemp seedling and leaf to produce transient transformants [134]. An agroinfiltration protocol (using *A. tumefaciens* strain EHA105) coupled with vacuum infiltration resulted in successful transient transformation of cotyledons and true leaves of young Cannabis seedlings [135]. A more efficient transformation of mature leaves, flowers, stem and root tissues using similar methods of agroinfiltration concluded that *A. tumefaciens* strain GV3101 is 1.7 times more efficient than strains EHA105 and LBA4404 [136]. Additionally, the transient transformation of Cannabis protoplast using PEG-mediated transformation was also recently reported to be effective in studying gene functions [137]. In another study, a nanoparticle-mediated transformation was successful in delivering soybean genes into Cannabis leaf tissues using silicon-dioxide-coated gold nanoparticles [138]. Together, these studies provided important protocols for Cannabis transient transformation that do not require going through the indirect organogenesis procedures that have been a bottleneck in genetic engineering studies. Although transient transformation can be effectively applied to study gene function in Cannabis, establishment of stable transformation is still required for genetic analysis of the progeny. A recent study reported a protocol for stable Agrobacterium-mediated transformation of Cannabis hypocotyls and cotyledons [11]. The study concluded that the rate of transformation and regeneration was higher in hypocotyl explants, but it varied among different Cannabis varieties. However, transformed explants were directly regenerated without going through the callus phase. In another recent study, a successful stable transformation using immature embryos followed by indirect organogenesis of their calli was reported [12]. To enhance the indirect regeneration potential, *Agrobacterium*-mediated transformation was used to overexpress endogenous developmental regulator genes which are known to have positive influences on shoot organogenesis. Moreover, the same study used CRISPR/Cas9 for the first time in Cannabis to edit the *phytoene desaturase* gene, resulting in gene-edited seedlings. Development of homozygous inbred lines of a crop with a desired trait is a tedious process that can take up to 10 generations by traditional methods [139]. Shortening of this breeding time is crucial for both scientific and commercial ends in crops such as Cannabis which have high commercial value. Doubled haploid (DH) technology can be employed for this purpose, as it has been used successfully in other crops. However, DH technology in Cannabis is yet to be fully developed.

### 8.2. Doubled Haploid

Doubled haploids (DH) are plants that carry genetic information from a single parent [140]. DH technology is used by plant breeders to rapidly make genetically homogeneous progenies in fewer generations than it takes in the traditional breeding. This technology has been used extensively in maize and other crops such as wheat, potato and sorghum [141]. To make haploid plants, breeders can use in vitro haploid gametophyte tissue culture, interspecific crossing or pollen irradiation procedures [140]. Once haploid plants are obtained, their chromosomes are doubled to make them fertile and robust by chemically blocking cell division without blocking chromosome duplication [140]. In Cannabis, interspecific crossing and pollen irradiation studies to make haploid plants have not been carried out, but studies to regenerate haploid plants directly by embryogenesis from microspores (androgenesis) have been conducted. In one study, a small percent (0.05-0.5%) of in vitro cultured hemp microspores formed embryo [10]. Recently, a study on two hemp varieties reported that cold-shock pretreatment of floral buds before in vitro culture increased the embryogenesis potential of the microspores [142]. The rate of embryo formation in the study (1.11 embryo formation for every 100 anthers), was also low and the microspore-derived embryo did not develop beyond 9 weeks. It has been reported that for successful embryogenesis, microspores have to be at, or just after, the first mitotic division when the cells are still in a uni-nucleate or early bi-nucleate stage [10]. Microspore culture at an appropriate developmental stage is therefore crucial but challenging. One other method to make haploid plants is the use of in planta gene-mediated haploid induction systems. This method entails crossing a line carrying the desired genetic information with a haploid inducer (HI) line [141]. In this crossing, the chromosomes from the HI will not be part of the embryo’s genome after fertilization. This haploid induction method has been used in plants such as *Zea mays,* where a HI carrying a *MATRILINEAL (MTL)/Patatin-Like Phopholipase A1 (ZmPLA1)/NOT LIKE DAD (NLD)* gene was used in a cross that resulted in haploid offspring [143]. Additionally, a mutation in the *Centromere histone H3 (CENH3)* gene has been reported to cause haploid induction in desired plants when these plants are crossed with an HI carrying this mutation [100,144]. Other genes that have been reported to have haploid induction ability are *DOMAIN OF UNKNOWN FUNCTION* 679 *membrane protein (DMP)* [145] and *indeterminate gametophyte1 (ig1)/LATERAL ORGAN BOUNDARIES (LOB*)-domain protein [146]. The molecular basis behind haploid-inducing ability of maize HI has been discovered [141,143]. This has allowed breeders to render haploid induction ability to plant lines through genetic engineering [144,145,147]. Although the use of HI has not yet been implemented in Cannabis, success in other plants indicate that this method can be used for haploid induction and DH studies. Once well established in Cannabis, DH technology can be useful in fixing desired characteristics and in reducing breeding generations required to obtain a desirable line. Being a plant with high variability in chemical and physical characteristics, as well as high commercial value, DH offers a promising way forward for Cannabis breeding. Moreover, researchers have made use of HI to deliver gene-editing tools into the egg cell of a plant through the methods called HI-Edit [148], and haploid-inducer-mediated genome editing (IMGE) [149]. These methods can also be used in gene editing procedures of Cannabis once well established. The fact that the HI genome, and the gene-editing machinery it carries, do not become part of the transformed embryo makes this technology appealing for commercial utilization of the engineered plants.

## 9. Conclusions

Cannabis research has been impacted by the decades-long criminalization of the plant. However, recent relaxation of restrictions has caused an increased interest in understanding the full potential of the plant. Its rapidly changing legal status, the growing market for its various products, and its potential use in bioenergy generation offer a glimpse into the plant’s future. However, research aiming at increasing production of the useful phytocannabinoids in planta and in heterologous systems are in their early stages. Moreover, conditions for fast and robust indirect regeneration of marijuana as well as of hemp are also not well established; consequently, efficient transformation studies are also limited. Nevertheless, the recent increase in research and publications on Cannabis and its propagation [44] is a sign that the efforts towards fulfilling the potential usefulness of this valuable plant will continue to accelerate. One of the areas that Cannabis researchers, particularly breeders, will be interested in is elucidation of the haploid-inducer genes in the plant. With the Cannabis genome database well established, studies on haploid induction through genetic engineering will speed up. The presence of genotypic variations within Cannabis subspecies [23], and the different response rates of these cultivars to transformation and to regeneration [11], remain a major challenge. Nevertheless, some of the haploid-inducing genes in different Cannabis cultivars have very few variations. For example, there is 99.1% protein identity between *Cannabis sativa DMP* of Finola (CsFN_05G0057910) and Jamaican Lion Dash (CsJLD_00G0583060) cultivars. This shows a promising possibility of making double haploids even in cultivars such as Cheungsam which do not have a publicly available genome database.

## Figures and Tables

**Table 1:** Harmful and beneficial effects of phytocannabinoid use.

**Health Conditions Caused by THC Use**	**References**
Psychosis	[82,83,84]
Memory impairment	[85,86]
Anxiety	[87,88]
Schizophrenia	[82,85,86,87,88,89]
Lack of attention	[88]
Interference with prenatal brain development	[85,88,90]
Impairment to psychomotor performance	[91,92]
**Health Conditions Treated by THC**	**References**
Pain	[93]
Multiple sclerosis	[94]
Glaucoma	[95]
Nausea	[81,93]
Loss of appetite for cancer and AIDS patients	[93]
Depression	[96]
Parkinson disease	[97]
Spasticity	[81]
Cancer	[98,99]
Tourette’s syndrome	[100]
**Health Conditions Treated by CBD**	**References**
Alzheimer’s disease	[101]
Pain	[93]
Multiple sclerosis	[94]
Depression	[96]
Schizophrenia	[102,103]
Cancer	[98,99]
Epilepsy	[79]

**Table 2:** Heterologous biosynthesis of phytocannabinoids.

Heterologous System Used	Enzyme Used	Precursor Molecules Supplied to System	Cannabinoids /Intermediate Compound Produced	References
Tobacco roots	THCAS	CBGA	THCA	[120]
*Pichia pastoris*	THCAS	CBGA	THCA	[121]
*Pichia pastoris*	THCAS	CBGA	THCA	[122]
*Escherichia coli*	THCAS	CBGA	None	[122]
*Saccharomyces cerevisiae*	Prenyltransferase (NphB) and THCAS	Olivetolic Acid and GPP	CBGA	[123]
*Pichia pastoris*	Prenyltransferase (NphB) and THCAS	Olivetolic Acid and GPP	THCA	[123]
*Saccharomyces cerevisiae*	Enzymes involved in biosynthesis of GPP, OA and phytocannabinoids	Galactose	THCA, CBDA, THCVA, CBDVA	[124]
*Saccharomyces cerevisiae*	Aromatic prenyltransferase (CsaPT4)	Glucose or glucose+hexanoic acid or glucose+OA	Olivetolic acid, CBGA	[9]
*Tobacco leaves*	Aromatic prenyltransferase (CsaPT4)	AAE1, OLS and OAC	OA glucoside	[9]

THCAS, tetrahydrocannabinolic acid synthase; CBGA, cannabigerolic acid; THCA, tetrahydrocannabinolic acid; GPP, geranyl diphosphate; CBDA, cannabidiolic acid; THCVA, tetrahydrocannabivarinic acid; CBDVA, cannabidivarinic acid; AAE1, acyl-activating enzyme 1; OLS, olivetol synthase; OAC, olivetolic acid cyclase; OA, olivetolic acid.

## Data Availability

Not applicable.

## References

[1] McPartland J.M. (2018). *Cannabis* Systematics at the Levels of Family, Genus, and Species. Cannabis Cannabinoid Res..

[2] Hillig K.W. (2005). Genetic evidence for speciation in *Cannabis* (Cannabaceae). Genet. Resour. Crop Evol..

[3] McPartland J.M., Hegman W., Long T. (2019). Cannabis in Asia: Its center of origin and early cultivation, based on a synthesis of subfossil pollen and archaeobotanical studies. Veg. Hist. Archaeobotany.

[4] Zhang Q., Chen X., Guo H., Trindade L.M., Salentijn E.M.J., Guo R., Guo M., Xu Y., Yang M. (2018). Latitudinal Adaptation and Genetic Insights Into the Origins of *Cannabis sativa* L.. Front. Plant Sci..

[5] Ren G., Zhang X., Li Y., Ridout K., Serrano-Serrano M.L., Yang Y., Liu A., Ravikanth G., Nawaz M.A., Mumtaz A.S. (2021). Large-scale whole-genome resequencing unravels the domestication history of *Cannabis sativa*. Sci. Adv..

[6] Deitch R. (2003). Hemp-American History Revisited: The Plant with a Divided History.

[7] Schultes R.E., Hofmann A., Ratsch C. (2001). Plants of the Gods-Their Sacred, Healing and Hallucinogenic Powers.

[8] Seddon T., Floodgate W. (2020). Regulating Cannabis: A Global Review and Future Directions.

[9] Gülck T., Booth J.K., Carvalho A., Khakimov B., Crocoll C., Motawia M.S., Møller B.L., Bohlmann J., Gallage N.J. (2020). Synthetic Biology of Cannabinoids and Cannabinoid Glucosides in *Nicotiana benthamiana* and *Saccharomyces cerevisiae*. J. Nat. Prod..

[10] Adhikary D., Kulkarni M., El-Mezawy A., Mobini S., Elhiti M., Gjuric R., Ray A., Polowick P., Slaski J.J., Jones M.P. (2021). Medical Cannabis and Industrial Hemp Tissue Culture: Present Status and Future Potential. Front. Plant Sci..

[11] Galán-Ávila A., Gramazio P., Ron M., Prohens J., Herraiz F.J. (2021). A novel and rapid method for Agrobacterium-mediated production of stably transformed *Cannabis sativa* L. plants. Ind. Crops Prod..

[12] Zhang X., Xu G., Cheng C., Lei L., Sun J., Xu Y., Deng C., Dai Z., Yang Z., Chen X. (2021). Establishment of an Agrobacterium -mediated genetic transformation and CRISPR/Cas9-mediated targeted mutagenesis in Hemp (*Cannabis sativa* L.). Plant Biotechnol. J..

[13] Small E., Cronquist A. (1976). A Practical and Natural Taxonomy for Cannabis. TAXON.

[14] McPartland J.M., Guy G.W. A question of rank: Using DNA barcodes to classify *Cannabis sativa* and *Cannabis indica*. Proceedings of the 24th Annual International Cannabinoid Research Society Symposium on the Cannabinoids.

[15] Kress W.J., Erickson D.L. (2007). A Two-Locus Global DNA Barcode for Land Plants: The Coding rbcL Gene Complements the Non-Coding trnH-psbA Spacer Region. PLoS ONE.

[16] Oh H., Seo B., Lee S., Ahn D.-H., Jo E., Park J.-K., Min G.-S. (2015). Two complete chloroplast genome sequences of *Cannabis sativa* varieties. Mitochondrial DNA Part A.

[17] Gilmore S., Peakall R., Robertson J. (2007). Organelle DNA haplotypes reflect crop-use characteristics and geographic origins of *Cannabis sativa*. Forensic Sci. Int..

[18] Lawrence R.G. (2019). Pot in Pans: A History of Eating Cannabis.

[19] Hillig K.W., Mahlberg P.G. (2004). A chemotaxonomic analysis of cannabinoid variation in *Cannabis* (Cannabaceae). Am. J. Bot..

[20] Anderson C.L. (1980). Leaf variation among Cannabis species from a controlled garden. Botanical Museum Leaflets.

[21] Cascini F., Farcomeni A., Migliorini D., Baldassarri L., Boschi I., Martello S., Amaducci S., Lucini L., Bernardi J. (2019). Highly Predictive Genetic Markers Distinguish Drug-Type from Fiber-Type *Cannabis sativa* L.. Plants.

[22] Fischedick J.T., Hazekamp A., Erkelens T., Choi Y.H., Verpoorte R. (2010). Metabolic fingerprinting of *Cannabis sativa* L., cannabinoids and terpenoids for chemotaxonomic and drug standardization purposes. Phytochemistry.

[23] Watts S., McElroy M., Migicovsky Z., Maassen H., van Velzen R., Myles S. (2021). Cannabis labelling is associated with genetic variation in terpene synthase genes. Nat. Plants.

[24] Grassi G., McPartland J.M., Chandra S., Lata H., ElSohly M. (2017). Chemical and morphological phenotypes in breeding of *Cannabis sativa* L.. Cannabis sativa L. Botany and Biotechnology.

[25] McPartland J.M., Small E. (2020). A classification of endangered high-THC Cannabis (*Cannabis sativa* subsp. indica) domesticates and their wild relatives. PhytoKeys.

[26] Fournier G., Richez-Dumanois C., Duvezin J., Mathieu J.-P., Paris M. (1987). Identification of a New Chemotype in *Cannabis sativa*: Cannabigerol—Dominant Plants, Biogenetic and Agronomic Prospects. Planta Med..

[27] De Meijer E.P.M., Hammond K.M., Sutton A. (2009). The inheritance of chemical phenotype in *Cannabis sativa* L. (IV): Cannabinoid-free plants. Euphytica.

[28] Pacifico D., Miselli F., Carboni A., Moschella A., Mandolino G. (2008). Time course of cannabinoid accumulation and chemotype development during the growth of *Cannabis sativa* L.. Euphytica.

[29] Hesami M., Pepe M., Alizadeh M., Rakei A., Baiton A., Jones A.M.P. (2020). Recent advances in cannabis biotechnology. Ind. Crops Prod..

[30] Faeti V., Mandolino G., Ranalli P. (1996). Genetic diversity of *Cannabis sativa* germplasm based on RAPD markers. Plant Breed..

[31] Forapani S., Carboni A., Paoletti C., Moliterni V.M.C., Ranalli P., Mandolino G. (2001). Comparison of Hemp Varieties Using Random Amplified Polymorphic DNA Markers. Crop Sci..

[32] Datwyler S.L., Weiblen G.D. (2006). Genetic Variation in Hemp and Marijuana (*Cannabis sativa* L.) According to Amplified Fragment Length Polymorphisms. J. Forensic Sci..

[33] Rotherham D., Harbison S.A. (2011). Differentiation of drug and non-drug Cannabis using a single nucleotide polymorphism (SNP) assay. Forensic Sci. Int..

[34] Sawler J., Stout J.M., Gardner K.M., Hudson D., Vidmar J., Butler L., Page J.E., Myles S. (2015). The Genetic Structure of Marijuana and Hemp. PLoS ONE.

[35] Doh E.J., Lee G., Yun Y.-J., Kang L.-W., Kim E.S., Lee M.Y., Oh S.-E. (2019). DNA Markers to Discriminate *Cannabis sativa* L. ‘Cheungsam’ with Low Tetrahydrocannabinol (THC) Content from Other South Korea Cultivars Based on the Nucleotide Sequences of Tetrahydrocannabinolic Acid Synthase and Putative 3-Ketoacyl-CoA Synthase Genes. Evid.-Based Complement. Altern. Med..

[36] Fett M.S., Mariot R.F., Avila E., Alho C.S., Stefenon V.M., Camargo F.A.D.O. (2019). 13-loci STR multiplex system for Brazilian seized samples of marijuana: Individualization and origin differentiation. Int. J. Legal Med..

[37] Zhang J., Yan J., Huang S., Pan G., Chang L., Li J., Zhang C., Tang H., Chen A., Peng D. (2020). Genetic Diversity and Population Structure of Cannabis Based on the Genome-Wide Development of Simple Sequence Repeat Markers. Front. Genet..

[38] Russo E.B. (2007). History of Cannabis and Its Preparations in Saga, Science, and Sobriquet. Chem. Biodivers..

[39] Kovalchuk I., Pellino M., Rigault P., van Velzen R., Ebersbach J., Ashnest J.R., Mau M., Schranz M.E., Alcorn J., Laprairie R.B. (2020). The Genomics of Cannabis and Its Close Relatives. Annu. Rev. Plant Biol..

[40] Fleming M.P., Clarke R.C. (1998). Physical evidence for the antiquity of *Cannabis sativa* L.. J. Int. Hemp Assoc..

[41] Small E., Chandra S., Lata H., ElSohly M. (2017). Classification of *Cannabis sativa* L. in relation to Agricultural, Biotechnology and Recreational Utilization. Cannabis sativa L. Botany and Biotechnology.

[42] Long T., Wagner M., Demske D., Leipe C., Tarasov P.E. (2016). Cannabis in Eurasia: Origin of human use and Bronze Age trans-continental connections. Veg. Hist. Archaeobotany.

[43] Duvall C.S. (2019). The African Roots of Marijuana.

[44] Monthony A.S., Page S.R., Hesami M., Jones A.M.P. (2021). The Past, Present and Future of *Cannabis sativa* Tissue Culture. Plants.

[45] Vassilevska-Ivanova R. (2019). Biology and ecology of genus Cannabis: Genetic origin and biodiversity. In Vitro production of cannabinoids. Genet. Plant Physiol..

[46] Chandra S., Lata H., ElSohly M.A., Walker L.A., Potter D. (2017). Cannabis cultivation: Methodological issues for obtaining medical-grade product. Epilepsy Behav..

[47] Adams T.K., Masondo N.A., Malatsi P., Makunga N.P. (2021). *Cannabis sativa*: From Therapeutic Uses to Micropropagation and Beyond. Plants.

[48] Hesami M., Baiton A., Alizadeh M., Pepe M., Torkamaneh D., Jones A.M.P. (2021). Advances and Perspectives in Tissue Culture and Genetic Engineering of Cannabis. Int. J. Mol. Sci..

[49] Slusarkiewicz-Jarzina A., Ponitka A., Kaczmarek Z. (2005). Influence of cultivar, explant source and plant growth regulator on callus induction and plant regeneration of *Cannabis sativa* L.. Acta Biol. Crac. Ser. Bot..

[50] Wielgus K., Luwanska A., Lassocinski W., Kaczmarek Z. (2008). Estimation of *Cannabis sativa* L. Tissue Culture Conditions Essential for Callus Induction and Plant Regeneration. J. Nat. Fibers.

[51] Lata H., Chandra S., Khan I.A., ElSohly M.A. (2010). High Frequency Plant Regeneration from Leaf Derived Callus of HighΔ9-Tetrahydrocannabinol Yielding *Cannabis sativa* L.. Planta Med..

[52] Hussain S.H.F. (2014). Cannabinoids production in *Cannabis sativa* L. An In Vitro Approach. Ph.D. Thesis.

[53] Movahedi M., Ghasemi-Omran V., Torabi S. (2014). The effect of different concentrations of TDZ and BA on in vitro regeneration of Iranian cannabis (*Cannabis sativa*) using cotyledon and epicotyl explants. J. Plant Mol. Breed..

[54] Hesami M., Jones A.M.P. (2021). Modeling and optimizing callus growth and development in *Cannabis sativa* using random forest and support vector machine in combination with a genetic algorithm. Appl. Microbiol. Biotechnol..

[55] Smýkalová I., Vrbová M., Cvečková M., Plačková L., Žukauskaitė A., Zatloukal M., Hrdlička J., Plíhalová L., Doležal K., Griga M. (2019). The effects of novel synthetic cytokinin derivatives and endogenous cytokinins on the in vitro growth responses of hemp (*Cannabis sativa* L.) explants. Plant Cell Tissue Organ Cult..

[56] Pepe M., Hesami M., Small F., Jones A.M.P. (2021). Comparative Analysis of Machine Learning and Evolutionary Optimization Algorithms for Precision Micropropagation of *Cannabis sativa*: Prediction and Validation of in vitro Shoot Growth and Development Based on the Optimization of Light and Carbohydrate Sources. Front. Plant Sci..

[57] Chaffey N. (2001). Hemp on the move. Trends Plant Sci..

[58] Callaway J.C. (2004). Hempseed as a nutritional resource: An overview. Euphytica.

[59] Shi G., Cai Q. (2009). Cadmium tolerance and accumulation in eight potential energy crops. Biotechnol. Adv..

[60] Ahmad R., Tehsin Z., Malik S.T., Asad S.A., Shahzad M., Bilal M., Shah M.M., Khan S.A. (2015). Phytoremediation Potential of Hemp (*Cannabis sativa* L.): Identification and Characterization of Heavy Metals Responsive Genes. Clean-Soil Air Water.

[61] Husain R., Weeden H., Bogush D., Deguchi M., Soliman M., Potlakayala S., Katam R., Goldman S., Rudrabhatla S. (2019). Enhanced tolerance of industrial hemp (*Cannabis sativa* L.) plants on abandoned mine land soil leads to overexpression of cannabinoids. PLoS ONE.

[62] Wang X., Li Q.X., Heidel M., Wu Z., Yoshimoto A., Leong G., Pan D., Ako H. (2021). Comparative evaluation of industrial hemp varieties: Field experiments and phytoremediation in Hawaii. Ind. Crops Prod..

[63] Montonya D. (2016). Hemp as Fibre and Food? Regulatory Developments and Current Issues.

[64] Kamat J., Roy D.N., Goel K. (2002). Effect of harvesting age on the chemical properties of hemp plants. J. Wood Chem. Technol..

[65] Robinson R. (1995). The Great Book of Hemp: The Complete Guide to the Environmental, Commercial and Medicinal Uses of the World’s Most Extraordinary Plant.

[66] Schluttenhofer C., Yuan L. (2017). Challenges towards Revitalizing Hemp: A Multifaceted Crop. Trends Plant Sci..

[67] Leyva D.R., McCullough R.S., Pierce G.N., Preedy V.R., Watson R.R., Patel V.B. (2011). Medicinal Use of Hempseeds (*Cannabis sativa* L.): Effects on Platelet Aggregation. Nuts and Seeds in Health and Diseases Prevention.

[68] World’s Top Exports (2019). Cannabis Oils Imports by Country. http://www.worldstopexports.com/Cannabis-oils-imports-by-country/.

[69] Technavio Report (2020). Legal Cannabis Market by Product and Geography—Forecast and Analysis 2020–2024.

[70] Finnan J., Styles D. (2013). Hemp: A more sustainable annual energy crop for climate and energy policy. Energy Policy.

[71] Das L., Liu E., Saeed A., Williams D.W., Hu H., Li C., Ray A.E., Shi J. (2017). Industrial hemp as a potential bioenergy crop in comparison with kenaf, switchgrass and biomass sorghum. Bioresour. Technol..

[72] Ren M., Tang Z., Wu X., Spengler R., Jiang H., Yang Y., Boivin N. (2019). The origins of cannabis smoking: Chemical residue evidence from the first millennium BCE in the Pamirs. Sci. Adv..

[73] UNODC (2019). World Drug Report. Cannabis and Hallucinogens.

[74] Jang J.H., Bae E.-K., Choi Y.-I., Lee O.R. (2019). Ginseng-derived patatin-related phospholipase PgpPLAIIIβ alters plant growth and lignification of xylem in hybrid poplars. Plant Sci..

[75] Jang J.H., Lee O.R. (2020). Patatin-Related Phospholipase AtpPLAIIIα Affects Lignification of Xylem in Arabidopsis and Hybrid Poplars. Plants.

[76] Jang J.H., Lee O.R. (2020). Overexpression of ginseng patatin-related phospholipase pPLAIIIβ alters the polarity of cell growth and decreases lignin content in Arabidopsis. J. Ginseng Res..

[77] Jang J.H., Seo H.S., Lee O.R. (2022). Overexpression of *pPLAIIIγ* in Arabidopsis Reduced Xylem Lignification of Stem by Regulating Peroxidases. Plants.

[78] Han Y., Bai Y., Zhang J., Liu D., Zhao X. (2020). A comparison of different oxidative pretreatments on polysaccharide hydrolyzability and cell wall structure for interpreting the greatly improved enzymatic digestibility of sugarcane bagasse by delignification. Bioresour. Bioprocess..

[79] WHO (2018). Cannabidiol (CBD) Critical Review Report. Expert Committee on Drug Dependence Fortieth Meeting.

[80] FDA (2020). FDA and Cannabis: Research and Drug Approval Process. https://www.fda.gov/news-events/public-health-focus/fda-and-Cannabis-research-and-drugapproval-process.

[81] NICE (2019). Cannabis-Based Medicinal Products. NICE Guideline. https://www.nice.org.uk/guidance/ng144/resources/Cannabisbased-medicinal-products-pdf-66141779817157.

[82] Arseneault L., Cannon M., Poulton R., Murray R., Caspi A., Moffitt T.E. (2002). Cannabis use in adolescence and risk for adult psychosis: Longitudinal prospective study. BMJ.

[83] Andréasson S., Allebeck P., Engström A., Rydberg U. (1987). Cannabis and schizophrenia. A longitudinal study of Swedish conscripts. Lancet.

[84] Barkus E., Murray R.M. (2010). Substance use in adolescence and psychosis: Clarifying the relationship. Annu. Rev. Clin. Psychol..

[85] Trezza V., Cuomo V., Vanderschuren L.J. (2008). Cannabis and the developing brain: Insights from behavior. Eur. J. Pharmacol..

[86] Gleason K.A., Birnbaum S.G., Shukla A., Ghose S. (2012). Susceptibility of the adolescent brain to cannabinoids: Long-term hippocampal effects and relevance to schizophrenia. Transl. Psychiatry.

[87] Renard J., Rushlow W.J., LaViolette S.R. (2018). Effects of Adolescent THC Exposure on the Prefrontal GABAergic System: Implications for Schizophrenia-Related Psychopathology. Front. Psychiatry.

[88] Lubman D.I., Cheetham A., Yücel M. (2015). Cannabis and adolescent brain development. Pharmacol. Ther..

[89] Solowij N., Yucel M., Lorenzetti V., Lubman D., Castle D., Murray R.M., D’Souza D.C. (2012). Does Cannabis cause lasting brain damage?. Marijuana and Madness.

[90] Grant K.S., Petroff R., Isoherranen N., Stella N., Burbacher T.M. (2018). Cannabis use during pregnancy: Pharmacokinetics and effects on child development. Pharmacol. Ther..

[91] D’Souza D.C., Perry E., MacDougall L., Ammerman Y., Cooper T., Wu Y.-T., Braley G., Gueorguieva R., Krystal J.H. (2004). The Psychotomimetic Effects of Intravenous Delta-9-Tetrahydrocannabinol in Healthy Individuals: Implications for Psychosis. Neuropsychopharmacology.

[92] Khiabani H.Z., Bramness J.G., BjøRneboe A., MøRland J. (2006). Relationship Between THC Concentration in Blood and Impairment in Apprehended Drivers. Traffic Inj. Prev..

[93] Croxford J.L. (2003). Therapeutic Potential of Cannabinoids in CNS Disease. CNS Drugs.

[94] Killestein J., Hoogervorst E.L.J., Reif M., Blauw B., Smits M., Uirdehaag B.M.J., Nagelkerken L., Polman C.H. (2003). Immunomodulatory effects of orally administered cannabinoids in multiple sclerosis. J. Neuroimmunol..

[95] Flach A.J. (2002). Delta-9-tetrahydrocannabinol (THC) in the treatment of end-stage open-angle glaucoma. Trans. Am. Ophthalmol. Soc..

[96] Turna J., Patterson B., Van Ameringen M. (2017). Is Cannabis treatment for anxiety, mood, and related disorders ready for prime time?. Depress. Anxiety.

[97] Frankel J.P., Hughes A., Lees A.J., Stern G.M. (1990). Marijuana for parkinsonian tremor. J. Neurol. Neurosurg. Psychiatry.

[98] Guzmán M. (2003). Cannabinoids: Potential anticancer agents. Nat. Rev. Cancer.

[99] Kalenderoglou N., MacPherson T., Wright K.L. (2017). Cannabidiol Reduces Leukemic Cell Size—But Is It Important?. Front. Pharmacol..

[100] Hesami M., Najafabadi M.Y., Adamek K., Torkamaneh D., Jones A.M.P. (2021). Synergizing Off-Target Predictions for In Silico Insights of CENH3 Knockout in Cannabis through CRISPR/Cas. Molecules.

[101] Watt G., Karl T. (2017). In vivo Evidence for Therapeutic Properties of Cannabidiol (CBD) for Alzheimer’s Disease. Front. Pharmacol..

[102] Leweke F.M., Piomelli D., Pahlisch F., Muhl D., Gerth C.W., Hoyer C., Klosterkötter J., Hellmich M., Koethe D. (2012). Cannabidiol enhances anandamide signaling and alleviates psychotic symptoms of schizophrenia. Transl. Psychiatry.

[103] Renard J., Norris C., Rushlow W., Laviolette S.R. (2017). Neuronal and molecular effects of Cannabidiol on the mesolimbic dopamine system: Implications for novel schizophrenia treatments. Neurosci. Biobehav. Rev..

[104] Bewley-Taylor D., Blickman T., Jelsma M. (2014). The Rise and Decline of Cannabis Prohibition: The History of Cannabis in the UN Drug Control System and Options for Reform.

[105] Bayer I., Ghodse H., Evolution of International Drug Control, 1945–1995 (1999). Bulletin on Narcotics. https://www.unodc.org/unodc/en/data-and-analysis/bulletin/bulletin_1999-01-01_1_page003.html.

[106] Musto D.F. (1972). The Marijuana Tax Act of 1937. Arch. General Psychiatry.

[107] United Nations Commission on Narcotic Drugs. CND Votes on Recommendations for Cannabis and Cannabis-Related Substances. Press Statement—. Vienna. https://www.unodc.org/res/commissions/CND/Mandate_Functions/scheduling-elearning-tutorial_html/Brochure_on_the_Scheduling_Procedures_under_the_International_Drug_Control_Conventions.pdf.

[108] ProCon (2021). Legal Recreational Marijuana States and DC. https://marijuana.procon.org/legal-recreational-marijuana-states-and-dc/.

[109] Eastwood N., Fox E., Rosmaria A. (2016). A Quiet Revolution: Drug Decriminalization Across the Globe. Release. Drugs, The Law & Human Rights. https://www.release.org.uk/sites/default/files/pdf/publications/A%20Quiet%20Revolution%20-%20Decriminalisation%20Across%20the%20Globe.pdf.

[110] MacKay R., Phillips K. (2016). The Legal Regulation of Marijuana in Canada and Selected Countries. Legal and Social Affairs Division, Parliamentary Information and Research Service.

[111] Abuhasira R., Shbiro L., Landschaft Y. (2018). Medical use of cannabis and cannabinoids containing products—Regulations in Europe and North America. Eur. J. Intern. Med..

[112] Areesantichai C., Perngparn U., Pilley C. (2020). Current cannabis-related situation in the Asia-Pacific region. Curr. Opin. Psychiatry.

[113] Schlag A.K. (2020). An Evaluation of Regulatory Regimes of Medical Cannabis: What Lessons Can Be Learned for the UK?. Med. Cannabis Cannabinoids.

[114] Tanney C.A.S., Backer R., Geitmann A., Smith D.L. (2021). Cannabis Glandular Trichomes: A Cellular Metabolite Factory. Front. Plant Sci..

[115] Gülck T., Møller B.L. (2020). Phytocannabinoids: Origins and Biosynthesis. Trends Plant Sci..

[116] Romero P., Peris A., Vergara K., Matus J.T. (2020). Comprehending and improving cannabis specialized metabolism in the systems biology era. Plant Sci..

[117] Andre C.M., Hausman J.-F., Guerriero G. (2016). *Cannabis sativa*: The Plant of the Thousand and One Molecules. Front. Plant Sci..

[118] Livingston S.J., Quilichini T.D., Booth J.K., Wong D.C.J., Rensing K.H., Laflamme-Yonkman J., Castellarin S.D., Bohlmann J., Page J.E., Samuels A.L. (2020). Cannabis glandular trichomes alter morphology and metabolite content during flower maturation. Plant J..

[119] Cascini F., Passerotti S., Martello S. (2011). A real-time PCR assay for the relative quantification of the tetrahydrocannabinolic acid (THCA) synthase gene in herbal Cannabis samples. Forensic Sci. Int..

[120] Sirikantaramas S., Morimoto S., Shoyama Y., Ishikawa Y., Wada Y., Shoyama Y., Taura F. (2004). The Gene Controlling Marijuana Psychoactivity: Molecular cloning and heterologous expression of Tetrahydrocannabinolic acid synthase from *Cannabis sativa* L.. J. Biol. Chem..

[121] Taura F., Dono E., Sirikantaramas S., Yoshimura K., Shoyama Y., Morimoto S. (2007). Production of Δ1-tetrahydrocannabinolic acid by the biosynthetic enzyme secreted from transgenic Pichia pastoris. Biochem. Biophys. Res. Commun..

[122] Zirpel B., Stehle F., Kayser O. (2015). Production of Δ9-tetrahydrocannabinolic acid from cannabigerolic acid by whole cells of Pichia (Komagataella) pastoris expressing Δ9-tetrahydrocannabinolic acid synthase from *Cannabis sativa* L.. Biotechnol. Lett..

[123] Zirpel B., Degenhardt F., Martin C., Kayser O., Stehle F. (2017). Engineering yeasts as platform organisms for cannabinoid biosynthesis. J. Biotechnol..

[124] Luo X., Reiter M.A., D’Espaux L., Wong J., Denby C.M., Lechner A., Zhang Y., Grzybowski A., Harth S., Lin W. (2019). Complete biosynthesis of cannabinoids and their unnatural analogues in yeast. Nature.

[125] Sirikantaramas S., Taura F., Tanaka Y., Ishikawa Y., Morimoto S., Shoyama Y. (2005). Tetrahydrocannabinolic Acid Synthase, the Enzyme Controlling Marijuana Psychoactivity, is Secreted into the Storage Cavity of the Glandular Trichomes. Plant Cell Physiol..

[126] Wilkinson S.T., Yarnell S., Radhakrishnan R., Ball S.A., D’Souza D.C. (2016). Marijuana Legalization: Impact on Physicians and Public Health. Annu. Rev. Med..

[127] Koltai H., Namdar D. (2020). Cannabis Phytomolecule ‘Entourage’: From Domestication to Medical Use. Trends Plant Sci..

[128] Matías-Hernández L., Jiang W., Yang K., Tang K., Brodelius P.E., Pelaz S. (2017). AaMYB1 and its orthologue AtMYB61 affect terpene metabolism and trichome development in *Artemisia annua* and *Arabidopsis thaliana*. Plant J..

[129] Sayre R., Soto-Aguilar M., Zidenga T., Goncalves E.C. (2020). Systems and Methods for Enhancing Trichome Formation and Density in Cannabis. U.S. Patent.

[130] Parsons J.L., Martin S.L., James T., Golenia G., Boudko E.A., Hepworth S.R. (2019). Polyploidization for the Genetic Improvement of *Cannabis sativa*. Front. Plant Sci..

[131] Huang S., Weigel D., Beachy R.N., Li J. (2016). A proposed regulatory framework for genome-edited crops. Nat. Genet..

[132] Feeney M., Punja Z.K. (2003). Tissue culture and Agrobacterium-mediated transformation of hemp (*Cannabis sativa* L.). In Vitro Cell. Dev. Biol. Plant.

[133] Wahby I., Caba J.M., Ligero F. (2013). Agrobacterium infection of hemp (*Cannabis sativa* L.): Establishment of hairy root cultures. J. Plant Interact..

[134] Schachtsiek J., Hussain T., Azzouhri K., Kayser O., Stehle F. (2019). Virus-induced gene silencing (VIGS) in *Cannabis sativa* L.. Plant Methods.

[135] Sorokin A., Yadav N.S., Gaudet D., Kovalchuk I. (2020). Transient expression of the *β-glucuronidase* gene in *Cannabis sativa* varieties. Plant Signal. Behav..

[136] Deguchi M., Bogush D., Weeden H., Spuhler Z., Potlakayala S., Kondo T., Zhang Z.J., Rudrabhatla S. (2020). Establishment and optimization of a hemp (*Cannabis sativa* L.) agroinfiltration system for gene expression and silencing studies. Sci. Rep..

[137] Beard K.M., Boling A.W.H., Bargmann B.O.R. (2021). Protoplast isolation, transient transformation, and flow-cytometric analysis of reporter-gene activation in *Cannabis sativa* L.. Ind. Crops Prod..

[138] Ahmed S., Gao X., Jahan M.A., Adams M., Wu N., Kovinich N. (2021). Nanoparticle-based genetic transformation of *Cannabis sativa*. J. Biotechnol..

[139] Prigge V., Xu X., Li L., Babu R., Chen S., Atlin G.N., Melchinger A.E. (2012). New Insights into the Genetics of *in Vivo* Induction of Maternal Haploids, the Backbone of Doubled Haploid Technology in Maize. Genetics.

[140] Gilles L.M., Martinant J.-P., Rogowsky P., Widiez T. (2017). Haploid induction in plants. Curr. Biol..

[141] Jacquier N.M.A., Gilles L.M., Pyott D.E., Martinant J.-P., Rogowsky P.M., Widiez T. (2020). Puzzling out plant reproduction by haploid induction for innovations in plant breeding. Nat. Plants.

[142] Galán-Ávila A., García-Fortea E., Prohens J., Herraiz F.J. (2021). Microgametophyte Development in *Cannabis sativa* L. and First Androgenesis Induction Through Microspore Embryogenesis. Front. Plant Sci..

[143] Kelliher T., Starr D., Richbourg L., Chintamanani S., Delzer B., Nuccio M.L., Green J., Chen Z., McCuiston J., Wang W. (2017). MATRILINEAL, a sperm-specific phospholipase, triggers maize haploid induction. Nature.

[144] Ravi M., Chan S.W.L. (2010). Haploid plants produced by centromere-mediated genome elimination. Nature.

[145] Zhong Y., Liu C., Qi X., Jiao Y., Wang D., Wang Y., Liu Z., Chen C., Chen B., Tian X. (2019). Mutation of ZmDMP enhances haploid induction in maize. Nat. Plants.

[146] Kermicle J.L. (1969). Androgenesis Conditioned by a Mutation in Maize. Science.

[147] Liu C., Li X., Meng D., Zhong Y., Chen C., Dong X., Xu X., Chen B., Li W., Li L. (2017). A 4-bp Insertion at ZmPLA1 Encoding a Putative Phospholipase A Generates Haploid Induction in Maize. Mol. Plant.

[148] Kelliher T., Starr D., Su X., Tang G., Chen Z., Carter J., Wittich P.E., Dong S., Green J., Burch E. (2019). One-step genome editing of elite crop germplasm during haploid induction. Nat. Biotechnol..

[149] Wang B., Zhu L., Zhao B., Zhao Y., Xie Y., Zheng Z., Li Y., Sun J., Wang H. (2019). Development of a Haploid-Inducer Mediated Genome Editing System for Accelerating Maize Breeding. Mol. Plant.

